# Hydrochlorate of Cocaine

**Published:** 1885-03

**Authors:** M. C. Armstrong

**Affiliations:** Brookville, Ind.


### Hydrochlorate of Cocaine.

BY M. C. ARMSTRONG, BROOKVILLE, IND.

Much has been said in dental journals recently, both pro and con, regarding the use of the new anaesthetic—Hydrochlorate of Cocaine—in dental surgery. While a few members of the profession have been unsuccessful in its use, it is my experience that beneficial results follow when used properly. Within the past few weeks I have tested its qualities as an ansæthetic in scores of cases, in every one of which it has proven entirely satisfactory.

There appears to be no pain in extracting, and only a slight sensation of pain after the tooth is drawn. In sensitive dentine I have found its use all that could be desired.

My method of using cocaine has been as follows: With a hypodermic syringe I inject two or three drops of the four per cent. solution into the gums on either side of the tooth, slipping the needle as far as possible. After four minutes have elapsed, another similar application is made, when the gum is dried and cocaine applied to the outside with a camel's hair brush. At the end of two minutes more, making in all ten minutes from the first injection, the gum lancet is used, and the tooth extracted, the whole operation being painless. By saturating a bit of cotton with the four per cent. solution, and inserting it in the cavity of a sensitive tooth I have been able to use the dental engine without causing pain. In all my experience with cocaine, I have yet to find a case in which it has not been effective. I have tried it on all kinds of teeth, from those which were ulcerated, decayed, or broken off, and from which the patient was suffering great pain, to those which were sound, and the results have been the same—painless. With the more fortunate brethren of the profession, I cry "Eureka." We are for painless dental surgery; the people demand it, and in cocaine, properly administered, they have it.

